# *Ex vivo* lung perfusion: recent advancements and future directions

**DOI:** 10.3389/fimmu.2025.1513546

**Published:** 2025-02-24

**Authors:** Kentaro Nakata, Isaac S. Alderete, Benjamin A. Hughes, Matthew G. Hartwig

**Affiliations:** ^1^ Duke Ex Vivo Organ Laboratory, Department of Surgery, Duke University Medical Center, Durham, NC, United States; ^2^ Division of Cardiovascular and Thoracic Surgery, Department of Surgery, Duke University Medical Center, Durham, NC, United States

**Keywords:** lung transplantation, organ preservation, machine perfusion, ex-vivo lung perfusion, donor lung assessment, ischemia-reperfusion injury, graft rehabilitation

## Abstract

Ex-vivo lung perfusion (EVLP) has emerged as a transformative technique in lung transplantation, offering a solution for evaluating and rehabilitating donor lungs that would otherwise be deemed unsuitable. This review article examines the significant advancements in EVLP technology and its application in clinical practice. We discuss the criteria for selection and rehabilitation of donor lungs, emphasizing the use of EVLP for lungs with compromised function due to factors like prolonged ischemic time and donor smoking history. Further, we elaborate on the technological advancements that have improved the functional assessment of lungs, including the development of more sophisticated perfusion solutions and the integration of artificial intelligence for real-time assessment. Additionally, we discuss the future prospects of EVLP, focusing on potential innovations in perfusion solutions, the integration of regenerative medicine and gene therapy to improve allograft quality. Through this comprehensive review, we aim to provide a clear understanding of the current status of EVLP and its promising future directions, ultimately contributing to improved outcomes in lung transplantation.

## 1 Introduction

Lung transplantation (LTx) is the preferred therapy for patients with end-stage lung disease, offering enhanced survival and improved quality of life compared to remaining on the waitlist (1). However, the demand for donor lungs consistently outstrips the available supply, and stringent regulatory oversight impedes innovation, leading to unfavorable outcomes for many candidates who either succumb to their condition or are removed from the waitlist before transplantation. It is estimated that only 15-25% of available lungs are ultimately utilized for transplantation following a meticulous evaluation, with the remainder either discarded or allocated for research purposes (2, 3). Donor lungs often referred to as “extended criteria” are increasingly contributed from older donors, donation after cardiac death (DCD), individuals with a significant history of smoking, or those with a PaO2/FiO2 ratio below 300, and offer an additional avenue to expand the donor pool (4). However, some studies have associated the utilization of these lungs with increased mortality and primary graft dysfunction (PGD), particularly in high-risk recipients, prompting certain groups to exercise caution in accepting such donor lungs. Nonetheless, recent research has demonstrated comparable outcomes when transplanting usable, albeit extended criteria, allografts (5, 6).

In the field of organ transplantation, traditional preservation methods have relied on static cold storage, maintaining donor lungs at temperatures between 0-4°C to minimize metabolic activity. While this approach facilitates stable storage, it precludes functional assessment of the organ. Given the persistent shortage of transplantable organs, transplant centers have increasingly considered grafts from extended-criteria donors that may require *ex situ* evaluation. Additionally, logistical complexities associated with multi-organ donation and competing clinical priorities may result in potentially viable organs going unused.

Recent advancements in ex-vivo lung perfusion (EVLP) technology have addressed these limitations by providing a platform for enhanced evaluation, extended preservation, and potential rehabilitation of donor lungs prior to transplantation. EVLP employs normothermic perfusion and ventilation techniques to maintain the organ’s metabolic functions, thereby enabling organ resuscitation and the potential administration of therapeutic agents.

This methodology represents a significant advancement in lung transplantation, offering improved assessment capabilities and the potential to expand the donor pool.

### 1.1 Indications for EVLP utilization

EVLP has revolutionized lung transplantation since its clinical introduction in 2001 by Steen et al. This innovative technique allows for the assessment and potential reconditioning of donor lungs that might otherwise be deemed unsuitable for transplantation, significantly expanding the donor pool. Primary indications are below;

#### 1.1.1 Impaired Oxygenation

Lungs with a low P/F ratio below the accepting center’s threshold (typically 250-350 mmHg) indicate compromised oxygen exchange.

#### 1.1.2 Pulmonary Edema

Presence of fluid accumulation observed on chest imaging. EVLP can assess the extent of edema and monitor its resolution during perfusion.

#### 1.1.3 Poor Lung Compliance

Compromised airway compliance affecting efficient lung expansion and contraction. EVLP enables assessment of lung mechanics and potential improvement during perfusion.

#### 1.1.4 High-Risk Donor History

- Extensive Blood Transfusion: Donors requiring massive transfusion during resuscitation.
- Aspiration: Suspected or confirmed aspiration of gastric contents or other materials.

#### 1.1.5 Donation After Cardiac Death (DCD)

- EVLP provides additional assessment time for DCD lungs, which may have uncertain quality due to warm ischemic time.

#### 1.1.6 Logistics

On occasion, donor lungs require procurement prior to allocation completion and the use of EVLP can extend the preservation time allowing for the allocation process to be completed.

There are additional considerations like prolonged ischemic time >6 hours, donors with sepsis and persistent atelectasis. Grafts considered transplantable, but failed to meet the primary criteria with these conditions are frequently preserved by EVLP. Advanced static hypothermic storage devices (i.e. LungGuard and BaroGuard) offer controlled hypothermic preservation at a temperature warmer than 4°C for lung preservation during transport (7), potentially reducing the need for EVLP in certain logistically concerned cases. In the case of BaroGuard, the airway pressure of the donor lung is also maintained throughout storage at 15 cm H2O. However, EVLP still plays a critical role in assessing lung function, particularly in borderline grafts, as it allows for active perfusion and evaluation before transplantation. There is still a discussion for specific criteria and in its present iteration, EVLP has become a crucial instrument for clinicians to evaluate organs with uncertain viability for *in vivo* use.

### 1.2 Clinical available platforms and EVLP techniques

Initial broad clinical experience of EVLP was described by the Toronto Lung Transplant Program. In this early uncontrolled trial, donors considered as “high risk” received EVLP for up to 4 hours. Based on the parameters during EVLP, donor lungs identified as suitable were selected for transplantation. Twenty-three lungs underwent *ex situ* assessment, of which 20 were utilized for transplantation. Compared to 116 lungs selected for transplantation and received standard cold storage, patients who received EVLP treated lungs showed comparable early outcomes in PGD at 72 hours after LTx and 30-day mortality (8).

In the United States, two clinically available lung perfusion systems are the XVIVO Perfusion System (XPS) and the TransMedics Lung Organ Care System (OCS). Lung Bioengineering provides centralized EVLP services using either the Toronto Protocol or the XPS. All commercially available platforms demonstrated primary effectiveness and safety in clinical trials. The effectiveness and safety of the OCS platform were showcased in the randomized, controlled, INSPIRE trial, enrolling 370 patients, with 151 receiving OCS-treated grafts out of 320 lung transplants. While short-term survival was comparable, the incidence of early Grade 3 PGD was lower in the OCS cohort (9). In another multicentered prospective international clinical trial, known as EXPAND, 93 extended criteria lungs were perfused and 79 were transplanted, resulting in an 87% utilization rate of donor lungs, even though they didn’t meet ideal transplantation criteria (10). The XPS system was studied in the multicenter prospective NOVEL trial, where initially unaccepted lungs underwent EVLP. Out of 216 donor lungs receiving EVLP, 110 were transplanted. EVLP-evaluated lungs demonstrated similar rates of PGD Grade 3 at 72 hours and comparable one-year survival to 116 retrospectively collected contemporary control lungs utilized without EVLP (11).

There are several important differences between the XPS and Lung OCS systems. These include the portability of the Lung OCS compared to the stationary nature of the XPS. There are also differences in the perfusion pumps, ventilators, pulmonary venous drainage, perfusion solutions and other details. Although there are many differences in the details of the devices, the general concepts remain the same. Ventilation in both systems is achieved using a mechanical ventilator connected to the endotracheal tube inserted into the trachea. A perfusate is circulated through the circuit, which is connected for antegrade flow to the pulmonary artery (PA). The Lung OCS allows for open pulmonary venous drainage, while the XPS and Toronto Protocol utilize a pulmonary venous cannula for a closed drainage system. To achieve oxygenation, a membrane gas exchanger is employed, and a leukocyte filter is frequently connected to remove leukocytes before the perfusate enters the PA. Normothermic (i.e., 37°) temperature control is maintained with a heater-cooler unit. After circulating through the lungs, the perfusate is collected in a volume reservoir from where it recirculates through the system using the same pump.

### 1.3 XVIVO Perfusion System (XPS)

The XPS received FDA approval for commercial use in 2019 and is a stationary perfusion system. Lungs are initially procured using static cold preservation and are subsequently transported to the recipient hospital or a centralized center, where they are connected to the XPS for EVLP. This system provides stable perfusion for up to 6 hours.

The XPS comprises the base components mentioned earlier, with notable features such as a centrifugal pump and a monitoring screen. The XPS utilizes acellular STEEN solution as a perfusate base, with potential additives like steroids, antimicrobials, and heparin. The XPS follows closely the Toronto protocol for EVLP, which begins with the lungs immersed in a cold storage preservation solution. The surgical team assesses the lungs for any injuries sustained during transport. Two cannulas, one connected to the LA and another to the PA, both equipped with built-in pressure sensors for monitoring, are attached. The lungs are then transferred to the XVIVO chamber, where the PA cannula is connected to the circuit, and anterograde flow is initiated with the perfusate at room temperature. The perfusate temperature is gradually increased to 37°C, with mechanical ventilation commencing at 34°C. The perfusate flow rate is gradually increased to the target flow of 40% of the estimated donor cardiac output within 60 minutes of initiation. The gas mixture (86% N2, 8% CO2, 6% O2) is initiated at 1L/min and titrated to maintain an inflow perfusate pCO2 between 35 and 45 mm Hg. Lung recruitment maneuvers are performed hourly, with a peak airway pressure of 25 cmH2O and an O2 challenge performed. Bronchoscopic examination and radiographic imaging are conducted periodically.

### 1.4 TransMedics Organ Care System (OCS)

The Lung OCS is a portable system designed to minimize cold ischemic times during lung transportation. Contraindications for OCS use include moderate to severe traumatic lung injury or pleural defects. Severe contusions and pleural defects contribute to poor performance *ex situ*. Lungs are procured in the same manner as for static cold storage preservation, with OCS priming occurring before lung installation. The system is primed with 3 units of packed red blood cells mixed with 2L of OCS Lung solution, a low potassium dextran solution mixed with glucose. The OCS solution is mixed with 1 unit of multivitamins, 20 IU of insulin, 4 mg of Milrinone, 40 mEq of Sodium Bicarbonate, 200 mg of Ciprofloxacin, and 200 mg of Voriconazole. Once the perfusate temperature reaches 32°C, the lungs are added to the system, where they are perfused at a rate of 1.5 to 2.5 L/min, and the perfusate is gradually warmed to a temperature of 37°C. Ventilation begins once the system reaches 34°C and is initiated with a tidal volume of 6 to 7 mL/kg, a respiratory rate of 12 breaths/minute, positive end-expiratory pressure of 5 to 7 cm of H_2_O, and FiO_2_ of 21%. The EVLP system is then transported back to the recipient hospital, where final assessments are made. If the lungs are deemed suitable, they are flushed with cold perfusate. The characteristics of the XVIVO and OCS systems are outlined in the accompanying figure.

## 2 Assessment of lungs during EVLP

Donor lung grafts can face various risks such as inflammation and infection depending on the donor’s condition. A key advantage of utilizing EVLP is that it enables physiological evaluation of the graft under controlled conditions. This section will review the representative parameters used for graft assessment during EVLP. Additionally, preclinical attempts for graft evaluation during EVLP will be discussed.

### 2.1 Pulmonary gas exchange

The most important function of the lung is alveolar gas exchange and lung’s oxygenating capacity is a critical functional measure. There are two ways to evaluate lung oxygenation. One method is to calculate the difference of pO_2_ between outflow to inflow (ΔpO_2_). The other method uses an observation gas which does not contain oxygen to evaluate pO2. P/F ratio has been used to quantify the pulmonary gas exchange disfunction (12). To evaluate the active or maximal lung function increasing load to the lung is a method. In the Toronto protocol, tidal volume is increased to 10 ml/kg, FiO_2_ is increased to 100% and the respiratory rate is increased to 10 per minute for 10 minutes. In the TransMedics protocol, a continuous monitoring mode is initiated for 3 minutes, with the flow rate increased from 1.5-2.0 to 2.0-2.5 L/min. An observation gas containing no oxygen is used to immediately decrease the partial oxygen pressure in inflow blood from 90 mmHg to 60 mmHg, and the oxygenation of the outflow blood is then measured. A P/F ratio or ΔP/F ratio below 300 renders it ineligible for transplantation for all protocols, with the strictest criteria requiring over 400 (13–15). Instead of its importance as criteria, specific FiO_2_ is not determined to detect P/F ratio. The Cleveland Clinic group showed P/F ratios vary depending on the FiO_2_ during EVLP (16). Sakota et al. reported noninvasive optical SaO_2_ imaging system using porcine model during EVLP (17). Using that system they showed FiO_2_ 1.0 is suitable value for detecting unfunctional lung.

### 2.2 Lung compliance

Decreasing compliance oftentimes is associated with loss of surfactant, flooded alveoli and/or atelectasis. In EVLP, lung compliance is a calculated value using tidal volume divided by difference between peak airway pressure and positive end expiratory pressure (PEEP). PEEP is determined by ventilator setting usually as 5-7 cmH_2_O in EVLP. Usually, tidal volume is kept at 5-7 ml/kg during EVLP, so donor body size and their ideal body weight (IBW) are factors that affect apparent compliance. In our experience, focusing on changes in compliance rather than absolute values provides more accurate assessment of lung health and function and utilizing the donor’s IBW should more optimally protect the lungs from barotrauma while *ex situ*. In many criteria, stable compliance or less than 15-20% change compared to baseline is required instead of clear cut off value while one criterion suggested the cut off value of peak airway pressure is under 25 cmH_2_O (13, 18).

### 2.3 Pulmonary vascular resistance

Pulmonary vascular resistance (PVR) is a calculated value based on pulmonary artery pressure (PAP) and flow rate. The difference between mean PAP and left atrium pressure is divided by flow rate, followed by 80 times. In open atrium system devices like the OCS lung™, left atrium pressure is zero. During prolonged EVLP preservation, it is typical to observe an increase in pulmonary resistance. In general, stable or less than 15-20% change compared to baseline PVR or mean PAP is required for lung utilization (18–20). Some criteria suggest a cut off value for systolic PAP as less than 15-20 mmHg (13, 21). When PVR increases occur during perfusion, this is usually secondary to worsening pulmonary edema. Lung procurement followed by normothermic machine perfusion (NMP) potentially induces inflammation caused by ischemia-reperfusion injury (IRI) and possibly NMP itself. Furthermore, another possible cause of increasing PVR during EVLP is the scavenging of nitric oxide (NO) by hemolysis. Some of the current EVLP systems employ perfusate with concentrated red blood cells added. Plasma free hemoglobin released from damaged erythrocytes can strongly bind to the potent vasodilator nitrous oxide (NO), resulting in elevated PVR (22). Thus when using cellular perfusates, it is crucial to maintain the health and integrity of the erythrocytes to prevent hemolysis.

### 2.4 Lung edema

A combination of IRI, endothelial dysfunction, mechanical stress, and inflammatory responses increases vascular permeability and contributes to the development of lung edema during EVLP. Fluid building-up in the endotracheal tube is a clear sign of edema. To evaluate lung edema precisely some criteria require a “collapse” or “deflation” evaluation. During a deflation test, the ventilator is disconnected at peak inspiration and the lung is visually inspected for adequacy of exhalation (14). Clinically, it is common to employ the use of standard X-ray of the lungs. Some have studied and reported using CT and MRI technologies as additional evaluation tools (23). Others have argued that CT and MRI may be too sensitive and over identify parenchymal abnormalities. The XVIVO XPS is constructed to specifically accommodate X-ray imaging on the device. In addition to these imaging approaches, organ edema, observed as weight gain of the graft, is a reliable assessment for water accumulation. Other groups have focused on donor lung weights at the donor hospital and during perfusion is an important predictor of utilization and outcomes. The highest lungs weight quartile after EVLP are associated with higher rates of PGD grade 3 at 72 hours (21.1%), as well as longer intensive care unit and hospital stays (24). Some contend that a donor lung weight adjusted for donor size corresponds to extra vascular lung fluid and can be used for decision-making in regard to using or discarding donor lungs (24). To further advance weight evaluation, a real-time lung weight monitoring system has been developed. Utilizing this approach, researchers have been successful in evaluating lung quality within 40 minutes (25).

Furthermore, EVLP has emerged as a versatile platform for advanced diagnostic techniques, including ultrasound-based assessments of lung water (EVLW). A notable development in this area is the implementation of direct lung ultrasound evaluation, known as CLUE (DireCt Lung Ultrasound Evaluation). This scoring system quantifies EVLW during EVLP by measuring the percentage of B-lines. The CLUE score has been shown to correlate with key parameters such as lung weight, wet/dry ratio, and PaO2/FiO2 ratio, allowing for the identification of lungs that are most suitable for clinical transplantation (26).

### 2.5 Other parameters and further attempt

Considerable research has gone in to identify biomarkers of lung health over the years. None of these has routinely been employed clinically to date. Pro-and anti-inflammatory cytokine levels in perfusate and bronchoalveolar lavage have been extensively studied. The biological parameters including IL-6, IL-8 and IL-1β are associated with lungs that are more likely to be accepted (27, 28). Iskender et al. (2017) reported cytokine filtration during EVLP improved donor quality (29). Increasing lactate levels are commonly observed during EVLP due to reduced clearance. The lactate to pyruvate acid ratio provides an indicator of the lung quality during EVLP (30). In addition to that, pH of the perfusate is an important parameter. Presumably, the resultant acidosis results in vasoconstriction increasing PVR (31).

Another novel technique for lung assessment includes ex-vivo pulmonary artery angioscopy (EXPLORE) which enables diagnosis and treatment of pulmonary embolism (PE) using direct video assessment of the PA. Using this method 16 donor lungs were identified as being suspicious for having PE and in 5 cases PE was directly observed and removed before EVLP (32). Two of them were utilized for transplantation without early complications.

Current clinical evaluation remains somewhat objective, as the accepting physician needs to synthesize results of multiple different measured parameters, none of which independently determine usability. Typically, these include the oxygenation, the pulmonary compliance, the lung x-ray results, changes in weight, perfusate loss from the reservoir during the evaluation period, PVR, pH, etc. For the OCS INSPIRE and EXPAND clinical trials, acceptance decision was made by PF ratio, bronchoscopy, and transplanting surgeon’s accessment (9, 10). For the XPS NOVEL trial clinical utilization was defined by bronchoscopy, stability or improvement in physiological values including PVR, compliance and airway pressures and delta PaO_2_. Additional parameters include chest x-ray appearance and absence of consolidation or excessive bogginess by palpation (11).

There is a machine learning model to select suitable donor for transplantation based on the data obtained during EVLP. The model evaluates the *ex-situ* lung function based on both physiological and biological parameters (33). In this research they revealed delta PaO_2_ and static compliance were important parameters. In addition to that, they emphasized the importance of the pH of the perfusate. Presumably, the resultant acidosis results in vasoconstriction increasing PVR (31). While this research provides insights into the traditional experiential aspects employed by skilled surgeons in donor lung assessment, the machine learning model is not yet widely available for real-time clinical assessment in transplant centers.

## 3 Prolonged graft preservation using EVLP

While clinicians typically use EVLP for 3 to 6 hours to evaluate donor lungs, there are multiple reports in the literature of prolonged preservation times, including graft preservation for over 12 hours with subsequent clinical use. For example, one retrospective clinical trial demonstrated that prolonged EVLP preservation exceeding 12 hours, following the Toronto protocol, did not impact early post-transplant outcomes in a carefully selected group of lung grafts (31). Others have reported extended perfusion times of up to 16 hours using the Lung OCS for clinical transplantation without early complications (34, 35). Importantly, sub-analyses of the NOVEL XPS trial data demonstrated an association of extended cold ischemia post-EVLP with a risk for PGD and 1-year mortality (36). Yet, the authors are not able to determine why the post EVLP cold-ischemic time was extended. Prolonging the duration of non-ischemic organ preservation carries significant potential to expand geographical boundaries and mitigate lung wastage. Despite this promise, additional strides in technology will be essential to achieve optimal outcomes in this pursuit.

In studies involving large animals, successful lung preservation for a duration of three days has been demonstrated through the combination of 10°C cold storage and EVLP. This achievement underscores the potential for further extension of preservation times (37). Endogenous NO exerts potent vasodilatory, anti-inflammatory, and anti-apoptotic effects in the lung, however during the intricate process of transplantation NO production is significantly attenuated. A multicenter randomized pilot study demonstrated that the administration of gaseous NO could potentially extend the period of organ stability and enhance the well-being of donor lungs (38). As perfusion duration is prolonged, the accumulation of inflammatory cytokines, metabolic waste, and depletion of essential nutrients becomes a significant concern. A variety of methods are available to counteract these time-dependent shifts with varying degrees of success. To maintain perfusate homeostasis, the Toronto protocol exchanges 100 mL of circulated perfusate with fresh every 2 hours. An alternative method of perfusate reconditioning is hemodialysis (31). Hemodialysis is best applied to RBC-containing perfusates, but can be used for all protocols. Some research showed acceptable results using hemodialysis within 6 hours, while others have demonstrated that using hemodialysis over 24 hours presented harmful effects including increasing PVR and PA pressure. Clearly, additional study is warranted, however with appropriate modifications for the particular environment of EVLP, hemodialysis may be the most effective means of automated perfusate reconditioning (39–41). In addition to waste elimination, an EVLP protocol that continuously supplemented with total parenteral nutrition for prolonged machine perfusion showed promising results for organ preservation up to 24 hours (42). Dual perfusion through both the PA and the bronchial artery perfusion has also been described in preclinical small animal models and may be further beneficial for preserving lung function (43).

## 4 EVLP delivery of therapeutics

### 4.1 EVLP as a platform for targeted therapeutic delivery

As the clinical application of EVLP continues to grow, it is important to note additional key practical advantages of the platform for the focal delivery of therapeutics.

#### 4.1.1 Enhancing safety in therapeutic delivery using EVLP

Chief among these features is the minimization of off-target drug activity on other organs. Injurious side effects, particularly nephrotoxicity and hepatotoxicity, remain a significant impediment for the development and use of novel therapeutics. The mechanisms that drive drug-induced liver injury (DILI) are manifold, resulting from direct toxicity as drug accumulates in the liver, exposure to toxic metabolites, or direct stimulation of immune cells present in the liver (44). In the case of therapeutic vehicles like adenovirus, injury results from both accumulation in the liver with off-target gene expression as well as direct activation of innate and adaptive immune responses by the vector (45, 46). With more than 1000 drugs demonstrating some degree of DILI risk (47), addressing this concern is paramount during development of novel therapeutics, and one that is almost entirely circumvented by ex vivo perfusion systems. Indeed, eliminating DILI risk further expands the therapeutic window for these drugs, enabling the use of much higher effective doses if desired. With the advent of therapeutic biologics including antibody-based therapies, new concerns have arisen of off-target actions that are near-totally precluded if delivered in an *ex vivo* setting. For instance, two compelling preclinical reports evaluated antibodies directed against HMGB1 (48) and S100A8/A9 (49) respectively, observing significant reductions in measures of IRI using the hilar clamp model. It is reasonable to expect that systemic administration could elicit substantial undesired effects on peripheral organ systems, ergo pretreating grafts via EVLP represents an ideal scenario for delivery. In the case of viral-mediated gene therapies, *ex vivo* administration carries the added benefit of eliminating capsid sequestration by liver hepatocytes and Kupffer cells that typically occurs with intravenous dosing (50, 51).

#### 4.1.2 Optimizing dosing and resource utilization with EVLP

Furthermore, use of a standardizable platform like EVLP can simplify dosing and reduce the use of sometimes scarce drug supply. Estimated blood volumes for human adults vary within a range of 4000-5000 mL (52), as compared to the user-definable 1500-2000 mL of perfusate employed during EVLP. This lower dilutional volume necessitates less drug to achieve the same effective final concentration that, in the case of particularly expensive therapeutics like biologics, can trim costs of the platform and further justify clinical implementation. For instance, eculizumab, an antibody that reduces innate immune activation by inhibiting C5 activation, has shown significant promise in reducing atypical hemolytic-uremic syndrome after kidney transplantation (53) and recent reports suggest could be efficacious for reducing allograft rejection after lung transplantation (54). At nearly $30,000 a dose, ex vivo administration of biologics can both reduce the drug dose required and use extended perfusion time to achieve maximal drug effect before transplantation. Additionally, accumulating clinical and preclinical evidence suggests that exogenously administered recombinant club cell secretory protein (rCCSP) elicits notable anti-inflammatory actions, and appears particularly effective for ameliorating the sequelae of acute respiratory distress syndrome (ARDS) (55, 56). Based on these observations, attention has turned toward similarly applying rCCSP for the mitigation of acute and chronic inflammatory responses following lung transplantation (57, 58). For both antibody and recombinant protein therapies, EVLP represents an ideal platform for the careful titration and delivery of particularly expensive biologics prior to transplantation.

#### 4.1.3 EVLP for disease modeling and therapeutic evaluation

There is also significant potential value for EVLP as a dedicated investigational platform, both in modeling disease states as well as demonstrating therapeutic efficacy of novel treatments. For example, a recent report demonstrated this by inducing ARDS via lipopolysaccharide (LPS) administration in discarded human lungs attached to an EVLP circuit (59). Investigators then administered a novel therapeutic, BC1215, directly to the vascular and airway compartments, respectively, and tested the efficacy of the drug on pro-inflammatory cytokine release by periodic perfusate sampling and correlating these levels with changes in functional measures like P/F ratio. Due to the isolated nature of the organ, establishing causality between disease state, biomarker abundance, and drug action is much more robust with this approach than *in vivo* while maintaining clinical relevance. Additionally, considerable attention has been shifted to exploring models of DCD, including uncontrolled DCD, in hopes of greatly expanding the donor pool by rehabilitating warm ischemic injury sustained by these lungs. Studies have examined how severity of injury varies with warm ischemic time (60), as well as the efficacy of medications with putative therapeutic action in ameliorating IRI damage (61).

Furthermore, EVLP has served as a platform for the testing of anti-inflammatory reagents, such as Adenosine 2A (A2A) receptor agonists. Injection of an A2A receptor agonist directly into the EVLP perfusate has been shown to enhance lung function after prolonged cold preservation in discarded human lungs (62). Building off of this, A2A receptor agonists have also been shown to improve lung function in preclinical DCD models when administered during EVLP (63, 64). Collectively, these studies exemplify EVLP’s capability as a platform for modeling disease states and conducting drug investigations, surpassing what could be achieved with *in vitro* modeling alone.

### 4.2 EVLP-based gene therapy

Expanding the donor pool through the use of DCD grafts in tandem with abrogating ACR/CLAD are two particularly active areas of transplant research that have been reinvigorated with the advent of ex vivo gene therapy prior to implantation of a donor graft. As alluded to previously, EVLP is especially well-suited as a delivery platform for the administration of viral-mediated therapies.

#### 4.2.1 Targeting IRI, alloimmune injury, and CLAD: key applications of EVLP-delivered gene therapy

Among the disease states amenable to EVLP-delivered gene therapy IRI, alloimmune injury (i.e. acute cellular rejection), and CLAD, are important areas, that merit particular attention, as all three significantly impact quality and quantity of life following lung transplantation. One such strategy is the overexpression of IL-10 that has been studied by several groups. The protective effects of IL-10, particularly in IRI (65), are well described in small and large animal models, and IL-10 overexpression delivered via an adenovirus vector has shown promise in the context of lung transplantation (66, 67). Alternatively, silencing of Fas using small interfering RNA has shown promise in reducing lung inflammation in preclinical studies (68, 69), and could be amenable to virus-mediated expression. Regardless of the transgene employed, EVLP is uniquely poised to allow virus administration under controlled conditions, for a user-specified period of time, and in the absence of potentially interfering substances.

#### 4.2.2 Addressing transduction barriers in viral gene therapy

A significant hurdle for viral-mediated gene therapy, especially for adenoviruses, is the presence of pre-existing or interfering antibodies that bind capsids and diminish transduction efficiency (70). Estimates of seroprevalence for pre-existing neutralizing antibodies (NAb) in humans against standard serotypes (e.g. AAV1-9) vary from approximately 10% to as high as 60% (71–73), with general agreement among most studies that AAV2 exhibits the highest rates of pre-existing Nabs (72, 74). NAb positivity against the vector presently represents a primary criterion for exclusion from gene therapy studies, however alternative strategies for *in vivo* use are being explored to mitigate the effect of NAbs. For instance, depleting immunoglobulins from patient blood via plasmapheresis has been employed with reasonably good efficacy (75), and recent work has shown the endopeptidase imlifidase (IdeS), a cysteine protease derived from *Streptococcus* that cleaves IgG, effectively digests circulating anti-AAV antibodies, permitting gene transduction in previously seropositive non-human primates (76).

Several clinical trials have highlighted the toxicity risks associated with high-dose AAV gene therapy, which can provoke significant immune responses (77).While the EVLP system may mitigate the risk of systemic inflammation, introducing AAV during EVLP could still trigger local inflammation, capillary leak syndrome, and potentially ARDS (78). Although EVLP allows for close monitoring and intervention before transplantation, careful optimization of AAV dosage, the selection of less immunogenic capsids, and the use of targeted immunosuppressive strategies will be crucial for minimizing these risks.

Despite the promise of these approaches, EVLP inherently avoids these concerns of NAbs through the use of perfusates devoid of serum, the NAb-containing compartment of blood. Specifically, standard EVLP perfusates for normothermic perfusion are generally comprised of a dextran- or albumin-based solution, for example OCS Lung solution or Steen solution respectively, that in the case of the former is subsequently doped with enriched red blood cells to act as an oxygen carrier. NAbs can exert profound inhibitory effects on viral transduction, resulting in near-total elimination of expression in mice, macaque, and humans (79). Similarly, EVLP perfusates eliminate concerns of capsid-reactive leukocytes that could additionally reduce transgene expression (70).

#### 4.2.3 Targeting organ compartments with cell-specific serotypes using EVLP

EVLP also offers the advantage of targeting different compartments of the organ, potentially with different cell-specific serotypes.

##### 4.2.3.1 Airway delivery for broad expression in lung epithelial cells

Preclinical experiments have predominantly utilized airway delivery for gene therapy, as this achieves robust, broad expression in the lung, particularly the airway epithelial cells whose dysfunction is associated with a variety of disease states including cystic fibrosis.

##### 4.2.3.2 Serotype-specific tropism for targeting vascular endothelial cells

One caveat to this approach is the poor infection of vascular endothelium that is we and others observe differential serotype-specific tropism depending on the route of delivery. This phenomenon demonstrates the importance of deploying an appropriate vector to elicit expression in the desired population.

### 4.3 AAV-mediated gene therapy using EVLP

Despite the promising future for AAV-mediated gene therapy, use of this platform nevertheless requires the payload be comparatively small (e.g. <4.5 kb), necessitates a sometimes weeks-long induction period, and achieves low rates of persistent (e.g. >1 year) expression. Unfortunately, enduring gene modification systems like CRISPR-Cas9 or rapid-onset protein expression via direct mRNA delivery are largely incompatible with AAVs without significant, technically-challenging modifications. To address these challenges, a resurgence in nanoparticle development is underway building on decades-old work with lipid micelles to consistently generate particles whose size and packaging capability are user definable (80). The caveat to these advantages is the capacity for cell-specific targeting implicit to viral delivery. EVLP, then, is uniquely poised to leverage the desirable features of nanoparticle delivery with an inherently selective delivery platform.

#### 4.3.1 CRISPR complex packaging

Titrating nanoparticle (NP) size with payload packaging efficiency continues to be a challenge for *in vivo* use (80), however due to the inherent selectivity of EVLP, higher effective concentrations of NP can be loaded into the perfusate to compensate for diminished efficiency.

A particularly attractive feature of lipid NP (LNP)s is the markedly lower immunogenic profile relative to viral vector-based delivery that could permit repeated administration (81). This is especially advantageous for targeting high-turnover cell populations like airway epithelial cells to maintain therapeutic efficacy. For this approach specifically, redosing via airway delivery post-transplant is furthermore possible and merits additional investigation.

Despite lower immunogenicity, activation of innate immune responses are nevertheless an important consideration, particularly in lung (82).

#### 4.3.2 Direct mRNA delivery

The majority of gene therapy investigations are DNA-based to elicit sustained transgene production. Following capsid uncoating, viral vector-delivered single-stranded DNA enters the nucleus, undergoes second strand synthesis, and ultimately circularizes into episomes for mRNA transcription and eventually expression of the transgene protein product. While episomal transgene expression can be stable for months, and in some reports years, following infection (83), the latency to robust expression of protein can be days or weeks post-infection, although use of self-complementary gene inserts reduces this interval somewhat (84). As well as onset of expression, another complication for use of AAV-based modifications is the considerable difficulty in establishing a cost-effective production process for the therapeutic (85).

In light of these challenges, alternative strategies for fast and relatively inexpensive modification of protein expression *in vivo* are being explored via direct delivery of mRNA and RNAi systems. RNA-based approaches like mRNA achieve low-latency, high peak production since it does not require nuclear entry to generate production. Delivery and stability of these constructs has proven challenging, though, spurring development of novel nanoparticle-mediated strategies.

A recent study employed siRNA-mediated silencing of Timp1 and observed significant reductions in inflammatory response to LPS administration in mice (86).

Ongoing studies are exploring the possibility of direct electroporation of target organs to achieve therapeutic uptake, mRNA in particular, and EVLP is particularly well-positioned for deploying this method.

## 5 Present challenges and future opportunities for EVLP deployment

### 5.1 Cost concerns of EVLP platforms

A significant hurdle for the widespread adoption of EVLP is the necessary consideration of cost versus benefits. Especially for low- and middle-income countries, the cost problem is an overwhelming barrier to utilize EVLP clinically. There is a need to develop more cost effective devices and disposable parts. Present estimates of institutional expenditures associated with lung transplantation using standard criteria donors and cold storage preservation vary considerably, though consistently reported ranges fall between $124,242 and $204,215 (1, 87, 88). With the added complexity of EVLP, additional per-case direct cost is on average $40,000-50,000, however this appears, at least in part, to be defrayed by an increased volume of procedures, among other advantages (1, 87, 88). Indeed, Peel at al (88). reported comparable costs of procedures conducted prior and after the availability of EVLP that the authors attribute to other clinical benefits of EVLP including shorter ICU stays that further offset expenditures. Our own analysis similarly revealed a relatively modest increase in procedure costs with EVLP, but also found that leveraging the technique for use with extended criteria grafts resulted in comparable post-operative outcomes (1). Therefore, the overall costs to the healthcare system may be mitigated by treating more people with end-stage lung disease. It is difficult to understate that transformative potential of EVLP on expanding the donor pool to include extended criteria organs, particularly given that only ~25% of donor lungs are utilized for transplantation (89). While graft viability can vary among systems, studies consistently observe that EVLP effectively enhances short-term outcomes including reduced manifestation of PGD (90).

There are a host of other secondary, difficult to quantify, benefits of EVLP that extend well beyond primary measures like PGD. For instance, seminal work by Cypel et al. (2011) (8) showed that extended criteria lungs receiving EVLP could be stably maintained for a median procurement-to-implantation interval of 10 hours 54 versus 6 hours 10 minutes for standard cold storage (SCS) while also exhibiting comparable post-operative primary outcomes (e.g. 15% post-transplant PGD rate versus 30% for SCS). This observation is reinforced by a recent meta-analysis showing similar findings across a bevy of studies (91). Extended preservation also permits a number of substantial quality of life improvements including streamlined procedure scheduling, allowing surgeons to conduct transplants during more traditional operating hours, simplifying patient scheduling, and allowing rigorous graft evaluation and patient matching. Collectively, these reports demonstrate that the benefits of EVLP are multi-factorial and include difficult to quantify measures that nevertheless merit consideration.

Centralized donor care in specialized centers enhances quality through standardization and the accumulation of expertise, resulting in better outcomes and more consistent performance (92–94). It also reduces costs by enabling the shared use of equipment and resources. Advances in technology are transforming the transplantation landscape, including the establishment of donor centers and procurement companies (95). Moreover, collaboration with translational research institutes has the potential to further accelerate the application of technology in clinical lung transplantation.

## 6 Conclusion

EVLP represents a pivotal advancement in the field of lung transplantation, significantly enhancing the viability and utilization of donor lungs (96). The technology has progressed from its initial experimental stages to a robust clinical tool that addresses the limitations of traditional donor lung assessment. By enabling the evaluation and rehabilitation of marginal lungs, EVLP has the potential to expand the donor pool and improve transplant outcomes. Ongoing research and technological innovations continue to refine EVLP protocols, promising further enhancements in graft quality and patient survival rates. The integration of advanced perfusion solutions, regenerative medicine techniques, and artificial intelligence-driven assessments holds great promise for the future of EVLP. As we look ahead, the continued evolution of EVLP will undoubtedly play a crucial role in overcoming organ shortages and maximizing lung transplantation success.
